# Rationale and Design of the ISOLATION Study: A Multicenter Prospective Cohort Study Identifying Predictors for Successful Atrial Fibrillation Ablation in an Integrated Clinical Care and Research Pathway

**DOI:** 10.3389/fcvm.2022.879139

**Published:** 2022-06-30

**Authors:** Dominique V. M. Verhaert, Dominik Linz, Sevasti Maria Chaldoupi, Sjoerd W. Westra, Dennis W. den Uijl, Suzanne Philippens, Mijke Kerperien, Zarina Habibi, Bianca Vorstermans, Rachel M. A. ter Bekke, Rypko J. Beukema, Reinder Evertz, Martin E. W. Hemels, Justin G. L. M. Luermans, Randolph Manusama, Theo A. R. Lankveld, Claudia A. J. van der Heijden, Elham Bidar, Ben J. M. Hermans, Stef Zeemering, Geertruida P. Bijvoet, Jesse Habets, Robert J. Holtackers, Casper Mihl, Robin Nijveldt, Vanessa P. M. van Empel, Christian Knackstedt, Sami O. Simons, Wolfgang F. F. A. Buhre, Jan G. P. Tijssen, Aaron Isaacs, Harry J. G. M. Crijns, Bart Maesen, Kevin Vernooy, Ulrich Schotten

**Affiliations:** ^1^Department of Cardiology, Radboud Institute for Health Sciences, Radboud University Medical Centre, Nijmegen, Netherlands; ^2^Department of Cardiology, Cardiovascular Research Institute Maastricht, Maastricht University Medical Centre, Maastricht, Netherlands; ^3^Centre for Heart Rhythm Disorders, Royal Adelaide Hospital, The University of Adelaide, Adelaide, SA, Australia; ^4^Department of Biomedical Sciences, Faculty of Health and Medical Sciences, University of Copenhagen, Copenhagen, Denmark; ^5^Department of Cardiothoracic Surgery, Cardiovascular Research Institute Maastricht, Maastricht University Medical Center, Maastricht, Netherlands; ^6^Department of Physiology, Cardiovascular Research Institute Maastricht, Maastricht University, Maastricht, Netherlands; ^7^Department of Medical Imaging, Radboud Institute for Health Sciences, Radboud University Medical Center, Nijmegen, Netherlands; ^8^Department of Radiology and Nuclear Medicine, Cardiovascular Research Institute Maastricht, Maastricht University Medical Center, Maastricht, Netherlands; ^9^School of Biomedical Engineering and Imaging Sciences, King’s College London, London, United Kingdom; ^10^Department of Respiratory Medicine, Maastricht University Medical Center, Maastricht, Netherlands; ^11^Department of Anesthesiology, Maastricht University Medical Center, Maastricht, Netherlands; ^12^Department of Cardiology, Amsterdam University Medical Center (UMC), Amsterdam, Netherlands

**Keywords:** atrial fibrillation, catheter ablation, pulmonary vein isolation, atrial fibrillation ablation, study design, translational research, prediction model

## Abstract

**Introduction:**

Continuous progress in atrial fibrillation (AF) ablation techniques has led to an increasing number of procedures with improved outcome. However, about 30–50% of patients still experience recurrences within 1 year after their ablation. Comprehensive translational research approaches integrated in clinical care pathways may improve our understanding of the complex pathophysiology of AF and improve patient selection for AF ablation.

**Objectives:**

Within the “IntenSive mOlecular and eLectropathological chAracterization of patienTs undergoIng atrial fibrillatiOn ablatioN” (ISOLATION) study, we aim to identify predictors of successful AF ablation in the following domains: (1) clinical factors, (2) AF patterns, (3) anatomical characteristics, (4) electrophysiological characteristics, (5) circulating biomarkers, and (6) genetic background. Herein, the design of the ISOLATION study and the integration of all study procedures into a standardized pathway for patients undergoing AF ablation are described.

**Methods:**

ISOLATION (NCT04342312) is a two-center prospective cohort study including 650 patients undergoing AF ablation. Clinical characteristics and routine clinical test results will be collected, as well as results from the following additional diagnostics: determination of body composition, pre-procedural rhythm monitoring, extended surface electrocardiogram, biomarker testing, genetic analysis, and questionnaires. A multimodality model including a combination of established predictors and novel techniques will be developed to predict ablation success.

**Discussion:**

In this study, several domains will be examined to identify predictors of successful AF ablation. The results may be used to improve patient selection for invasive AF management and to tailor treatment decisions to individual patients.

## Introduction

The prevalence of atrial fibrillation (AF) has risen substantially over the past decade, and it continues to rise due to the aging population and the increasing rate of concomitant risk factors and underlying structural heart diseases (1). AF is associated with an increased risk of ischemic stroke, developing and worsening of heart failure, and a significant symptom and financial burden (2). To maintain sinus rhythm and to decrease symptoms related to AF, catheter ablation is recommended in symptomatic patients (3). However, despite advanced ablation techniques and improved ablation outcomes over the last decade, about 30–50% of patients still experience recurrences of atrial arrhythmias within 1 year after the procedure (4, 5).

Although several readily available characteristics (including demographic information, established clinical AF risk factors, and left atrial volume) have previously been identified as predictors for AF ablation success, it remains challenging to estimate the success rate for an individual patient (6). A range of novel techniques estimating the extent of the atrial AF substrate has been proposed: to aid in the prediction of individual success rates biomarkers, such as inflammatory mediators and markers for fibrosis (7, 8), common gene variants associated with AF, and non-invasive electrophysiological characteristics recorded on surface electrocardiograms (ECG) (9, 10). Additionally, a more detailed characterization of different AF patterns based on frequency, duration, and manner of conversion of AF episodes might further help to characterize the AF phenotype, although its predictive value for ablation success has not yet been described (11).

It is the aim of the multicenter prospective “IntenSive mOlecular and eLectropathological chAracterization of patienTs undergoIng atrial fibrillatiOn ablatioN (ISOLATION)” cohort study to systematically examine predictors of successful AF ablation in the following domains: (1) clinical factors, (2) AF patterns detected using rhythm monitoring devices, (3) anatomical characteristics, (4) electrophysiological characteristics, (5) circulating biomarkers, and (6) individual genetic background. Factors from these domains will be combined in a multimodality model for the prediction of ablation success, which may help to improve patient selection for invasive AF management. Herein, we outline the design of the ISOLATION study and describe how all study procedures are implemented into a standardized, integrated clinical care and research AF ablation pathway.

## Methods and Analysis

### Study Design

**FIGURE 1:**
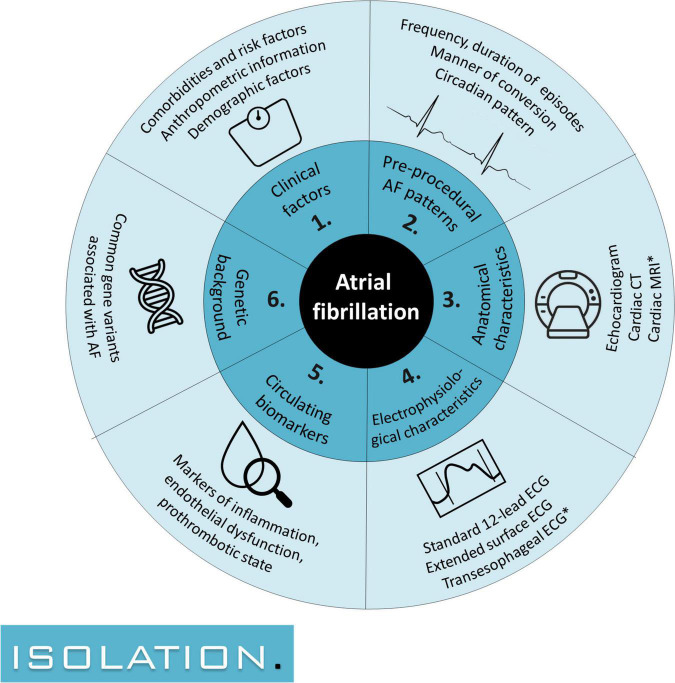
The 6 domains of interest in which predictors for successful atrial fibrillation ablation are sought: (1) clinical risk factors, (2) pre-procedural AF patterns, (3) anatomical characteristics, (4) electrophysiological characteristics, (5) circulating biomarkers, and (6) genetic background. AF, atrial fibrillation; CT, computed tomography; ECG, electrocardiogram; MRI, magnetic resonance imaging. Study procedures with an asterisk are conducted for a subset of patients.

The ISOLATION is a multicenter prospective cohort study designed to identify predictors of successful AF ablation in six different domains (Figure 1). The study was initiated in July 2020 and aims to include 650 patients scheduled for AF ablation in two Dutch university hospitals, the Maastricht University Medical Centre (MUMC) and the Radboud University Medical Centre (Radboudumc). Clinical characteristics, results of routine clinical tests (e.g., laboratory results, cardiovascular imaging) and several additional study procedures are collected before, during, and after the ablation. The study protocol was reviewed and approved by the ethics committee of the MUMC (METC azM/UM, NL70787.068.19) and is registered at clinicaltrials.gov (NCT04342312) and in the Netherlands Trial Register (NL7894).

### Study Population

Patients are eligible for participation in this study if they are 18 years of age or older, have documented AF, are scheduled for an AF ablation (first procedure or redo) in either participating center, and are able and willing to provide written informed consent. There are no restrictions for AF ablation modality nor for potential previous ablation procedures. Possible ablation modalities include percutaneous techniques (cryoballoon ablation or radiofrequency ablation), surgical ablations (epicardial ablation, concomitant ablation) or a combined procedure (hybrid ablation). Patients are excluded if they are deemed unfit to participate due to a serious medical condition or if they undergo an emergency procedure. In- and exclusion criteria are listed in Table 1.

**TABLE 1:** In- and exclusion criteria of the ISOLATION study.

**Inclusion criteria**	
1	18 years of age or older;
2	Documented atrial fibrillation;
3	Scheduled for atrial fibrillation ablation or redo atrial fibrillation ablation;
4	Able and willing to provide written informed consent.
**Exclusion criteria**	
1	Patients deemed unfit to participate due to a serious medical condition before ablation, as deemed by their treating physician;
2	Patients undergoing an emergency ablation procedure.

### Endpoints

The primary outcome measure is ablation success, defined as freedom from documented recurrence of atrial arrhythmia 12 months after the index procedure. Recurrences in the first 3 months are exempted (blanking period). Atrial arrhythmias are defined as AF, atrial tachycardia, or non-isthmus dependent atrial flutter. Episodes of atrial arrhythmia must be documented on an ECG, Holter monitoring (minimum duration of 30 seconds), or an implanted device (atrial high rate episode during at least 5 minutes or mode switch, confirmed as being AF or other atrial arrhythmia by a trained physician). To detect symptomatic and asymptomatic recurrences of atrial arrhythmias, 48-h Holter monitoring is performed at 6 and 12 months after the ablation procedure. Holter recordings may be extended or additional Holter recordings may be added when clinically indicated. 12-lead ECGs are obtained at 3 and 12 months, and patients are encouraged to have additional ECGs recorded if they experience symptoms between visits.

Key secondary outcomes include time to first recurrence of atrial arrhythmia or AF after the blanking period, freedom from documented recurrence of atrial arrhythmia at 24 months, redo procedures, disease progression to persistent or permanent AF, changes in circulating biomarkers, and changes in non-invasive electrophysiological markers for substrate quantification (Table 2).

**TABLE 2:** Primary and secondary endpoints in the ISOLATION study.

Primary endpoint
Ablation success, defined as freedom from documented recurrence of atrial arrhythmia after 12 months. Recurrences in the first 3 months after the index procedure (blanking period) are exempted.
**Secondary endpoints**
Time to recurrence of atrial arrhythmia after the blanking period;
Time to recurrence of AF after the blanking period;
Freedom from documented recurrence of atrial arrhythmia after 24 months. Recurrences in the blanking period are exempted;
Early AF recurrences, defined as any episode of AF during the blanking period;
Early recurrences of atrial arrhythmia, defined as any episode of AF, atrial tachycardia or non-isthmus dependent atrial flutter during the blanking period;
Disease progression to persistent or permanent AF;
Changes in circulating biomarkers and non-invasive electrophysiological markers for substrate quantification;
Use of antiarrhythmic drugs 1 year after ablation;
Redo procedures, defined as repeated ablation procedure with the goal to prevent recurrence of AF or reduce the AF burden after one or more previous attempts to achieve the same goal;
Number of veins with pulmonary vein reconnection at redo procedure;
Major adverse cardiovascular events.

### Study Enrollment

**FIGURE 2:**
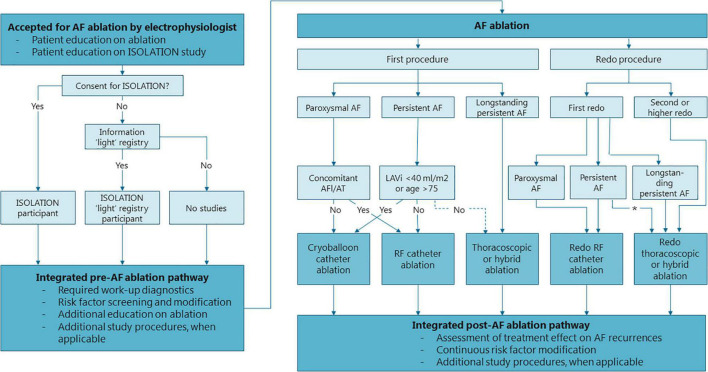
Flowchart of the standardized, integrated clinical care and research pre- and post AF ablation pathway. Structure of the pre- and post AF ablation pathway and general recommendations for the type of AF ablation. Treating physicians may choose to deviate from these recommendations depending on specific patient characteristics of patient preference. *Thoracocopic/hybrid ablation is strongly considered if LAVi >5− ml/m^2^ or in case of patient preference. AF, atrial fibrillation; LAVi, left atrial volume index; RF, radiofrequency.

Patients accepted for AF ablation receive verbal and written information about the ISOLATION study and are scheduled to visit a standardized AF ablation work-up pathway. Patients willing to participate provide written informed consent for ISOLATION prior to their visit at the work-up pathway, or during this pathway but prior to all study procedures. Patients that decline participation in the ISOLATION study are asked to participate in the “light” version of the study, the “Clinical electrophysiology registry MUMC + and Radboudumc” (ethical committee number 2019–1022). Participants in this ISOLATION “light” registry undergo the same standardized pre- and post AF ablation pathway, with the exception that most of the study procedures are omitted. Patients that do not wish to participate in either study complete the standardized pathway without any additional study procedures (Figure 2).

### Pre-ablation Work-Up

The standard work-up for ablation in the participating centers consists of a systematic collection of clinical information, vital signs, 12-lead ECG, blood tests, imaging to assess pulmonary vein anatomy (either computed tomography or cardiac magnetic resonance imaging), and screening by an anesthesiologist. Echocardiography is performed in patients without recent imaging or when recent imaging is of insufficient quality. During the work-up, patients are systematically screened for common comorbidities and triggers for AF such as hypertension, obesity, hyperlipidemia, diabetes mellitus, and chronic obstructive pulmonary disease (COPD) according to current AF guidelines (3). In addition, all patients without known obstructive sleep apnea (OSA) are referred for remote testing for sleep disordered breathing, as described previously in more detail (12). If any of the risk factors is present, applicable treatment is initiated parallel to the AF treatment.

**FIGURE 3:**
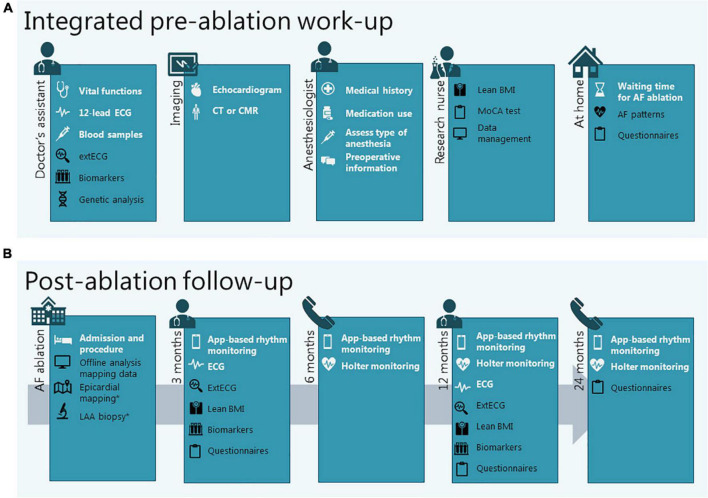
Integration of clinical diagnostics and study procedures in the work-up before **(A)** and the follow-up after **(B)** AF ablation. Procedures in white are standard clinical procedures, procedures in black are added for research purposes. *When applicable (in case of epicardial or hybrid ablation). AF, atrial fibrillation; BMI, body mass index; CMR, cardiac magnetic resonance imaging; CT, computed tomography; ECG, electrocardiogram; extECG, extended surface electrocardiogram; LAA, left atrial appendage; MoCa, Montreal Cognitive Assessment.

The pre-ablation preparation is structured in a care pathway at the outpatient clinic that allows the entire work-up to be completed in a single visit (Figure 3A, standard work-up procedures in white). All patients scheduled for AF ablation complete this pre-AF ablation work-up, regardless of study participation.

#### Study Procedures During Pre-ablation Work-Up

For ISOLATION study participants, the standard pre-AF ablation work-up is combined with the following baseline study procedures: determination of body composition, extended surface ECG (extECG), analysis of biomarkers and common gene variants, questionnaires, and characterization of AF patterns (Figure 3A, study procedures in black). For ISOLATION “light” registry participants the study procedures are limited to analysis of biomarkers, common gene variants, and questionnaires.

Both the clinical facets and research aspects of the pre-ablation work-up are overseen by a case manager. The case manager educates patients on the AF ablation procedure and study participation, coordinates study procedures, notifies abnormalities in results of diagnostic tests, and provides patients with information on logistics and planning.

### Atrial Fibrillation Ablation

AF ablation comprises pulmonary vein isolation (PVI) with or without additional lesions. The type of ablation that is performed is determined in a multidisciplinary team meeting and depends on the patient’s preference and characteristics. The choice of ablation strategy is not influenced by study participation. In the participating centers, first ablations for paroxysmal AF and for persistent AF with normal to modestly dilated atria or in elderly patients are usually performed by cryoablation (Figure 2). First ablations for persistent AF with advanced atrial dilation or with concomitant atrial flutter or atrial tachycardia are mostly performed by wide atrial circumferential ablation (WACA) using radiofrequency applications, if needed complemented with cavo-tricuspid isthmus ablation (in case of typical atrial flutters). Patients with longstanding persistent AF (> 1 year) are usually treated with thoracoscopic or hybrid AF ablation. Patients with recurrent AF undergoing a second (redo) procedure are mainly treated with radiofrequency ablation or thoracoscopic/hybrid AF ablation, whereas for third or further procedures hybrid procedures are recommended, if patients are suitable candidates and no earlier hybrid treatment was performed. Patients with an indication for cardiac surgery and known AF are discussed in an arrhythmia team and considered for concomitant epicardial AF ablation. Treating physicians may choose to deviate from these recommendations depending on specific patient characteristics and patient preference. All patients planned to undergo an endocardial procedure are treated with oral anticoagulants around the ablation (3). In addition, a transesophageal echocardiogram is performed prior to all endocardial procedures to exclude intracardiac thrombi.

#### Study Procedures During Atrial Fibrillation Ablation

Whether additional, optional study procedures are performed during the procedure depends on the type of ablation selected (Figure 3B). If endocardial electroanatomical mapping is performed during the ablation procedure, these data may be used for additional offline analyses. In the case of hybrid or concomitant epicardial ablation, additional epicardial mapping, and left atrial appendage (LAA) biopsies may be performed (Supplementary Material 1).

### Post-ablation Follow-Up

After the ablation procedure, patients return to the outpatient clinic at 3 and 12 months and have scheduled teleconsultations after 6 and 24 months. Prior to the contacts at 6, 12, and 24 months, a 48-h Holter recording is performed. 12-lead ECGs are obtained during every on-site follow-up visit. In addition, patients receive an on-demand 7-day prescription for the use of the smartphone application FibriCheck (Qompium, Hasselt, Belgium) prior to all scheduled consultations. FibriCheck is an app that uses photoplethysmogram (PPG) signals to assess heart rate regularity and symptom-rhythm correlation (13). Patients are instructed to measure 3 times per day and in the case of symptoms, in accordance with the TeleCheck-AF approach (14). If irregularity of the heartbeat is detected and AF is suspected, additional or longer Holter recordings may be performed to confirm AF recurrence by ECG documentation (Figure 3B, standard follow-up procedures in white).

#### Study Procedures During Post-ablation Follow-Up

For ISOLATION participants, on-site follow-up visits are complemented with the following study procedures: determination of body composition, extECG, and analysis of biomarkers. Questionnaires are repeated at 3, 12, and 24 months after the ablation (Figure 3B, study procedures in black). For ISOLATION “light” registry participants, study procedures are limited to the questionnaires.

### Short Description of Study Procedures

The rationale for the different study procedures is described in Supplementary Material 1, along with detailed descriptions of the procedures. A condensed version is provided here.

#### Determination of Body Composition

To explore the correlation between different anthropometric measures and ablation success, weight, fat percentage and lean body mass indices are estimated using bioelectrical impedance measurements obtained with body composition monitors. The results of these measurements are part of domain 1: clinical factors (Figure 1).

### Pre-procedural Rhythm Monitoring

To gain more insight into pre-ablation AF patterns, patients awaiting the AF ablation procedure receive a handheld patient-operated device which records single-lead ECGs. Patients are asked to record their heart rhythm three times a day during a maximum of 4 weeks. Additional registrations are recorded at each onset or relief of arrhythmia symptoms. Symptoms and their correlation to ECG recordings are documented in a patient diary. The obtained AF patterns are studied in domain 2 (Figure 1).

### Extended Surface Electrocardiogram

Detailed analysis of P-wave and fibrillation wave (F-wave) features from surface ECGs are increasingly used to characterize the degree of electrophysiological changes in the atria. In this study, unfiltered extECGs using a total of 21 leads will be recorded for up to 5 min to allow for signal-averaged analyzation of P-waves (for patients in sinus rhythm) and for examination of F-wave frequency and complexity (for patients in AF). The results are a component of domain 4: electrophysiological characteristics (Figure 1).

### Blood Samples

Blood for biomarker analyses is drawn at three separate time points during the study. Genetic analysis is performed on the blood drawn at baseline. Biomarkers of interest include inflammatory mediators, markers of myocardial wall stress, markers of fibrosis, markers of endothelial dysfunction and pro-thrombotic state, and markers for expression of genes regulating electrophysiological characteristics. The results are studied in domain 5: circulating biomarkers and 6: genetic background (Figure 1).

### Questionnaires

The following questionnaires are completed: Montreal Cognitive Assessment (MoCA), Toronto AF Severity Scale (AFSS), Atrial Fibrillation Effect on QualiTy-of-life (AFEQT), STOP-Bang, and a combined COPD questionnaire. Additional information on the questionnaires is provided in Supplementary Material 1.

### Additional Procedures for Substudies

Specific subsets of patients may be eligible to participate in a substudy of the ISOLATION study and/or ISOLATION “light” registry. Current areas of interest include cardiovascular imaging, non-invasive and invasive electrophysiological characterization, and concomitant comorbidities. Substudies that are currently actively enrolling patients and their primary objectives are listed in Supplementary Material 2. Patients eligible for participation in a substudy are asked for additional consent.

### Data Management

Patient identifiers are removed from study data, biological samples and recordings and replaced with a unique study number. Coded data is entered in a password-protected, secure electronic CRF [Castor EDC]. Raw data of procedures (e.g., extECGs) are stored under the respective patient identifier and will be available for further offline analysis. All information generated in this study will be considered confidential and is handled in according to the General Data Protection Regulation and the Dutch Act on Implementation of the General Data Protection Regulation. Monitoring is performed by the Clinical Trial Center Maastricht (CTCM) in accordance with the predefined monitoring plan and prevailing guidelines.

### Statistical Considerations

Data analyses will be performed with SPSS Statistics, version 25.0 or higher (IBM SPSS Inc., Chicago IL). Baseline data will be presented by count (percentage) for categorical variables and compared using the Chi-square test. Continuous data will be presented as means (± standard deviation) or medians (interquartile range) and compared using the independent samples *t*-test or Mann-Whitney *U*-test. Missing data will be estimated using multiple imputation by chained equations. Uni- and multivariable regression analyses will be performed to analyze the relation of dependent variables with the primary and applicable secondary endpoints. The secondary endpoints time to recurrence of atrial arrhythmia and AF will be assessed using Cox proportional hazard regression. All endpoints will be assessed using a level of significance of α = 0.05.

#### Development of Prediction Model and Sample Size Calculation

An exploratory multimodality model will be created to predict the primary endpoint (ablation success). Ideally, to develop a reliable prediction model all variables of interest that will be studied in the model should be defined prior to the data collection. However, this study includes several relatively new techniques, which each provide a multitude of possible variables of interest for which the predictive value has not been established. Defining variables to be included in the model up-front could limit its eventual predictive value, as the predefined variables might not prove to be the most discriminative variables. Therefore, an exploratory analysis of variables in the following categories will be performed first (Figure 1): (1) clinical risk factors, (2) pre-procedural AF patterns, (3) anatomical characteristics, (4) electrophysiological characteristics, (5) circulating biomarkers, and (6) genetic background. For subcategory 1–5 the three variables with the strongest correlation to ablation success are selected and presented to the model. For subcategory 6 the two strongest predictors are used. The ablation technique that is chosen is used as a separate predictor. This approach leads to a total of 18 variables used to develop the multimodality model. To ensure sufficient power to explore these 18 variables, a total of 180 events is required to conform to the rule-of-thumb to aim for 10 events per variable. When assuming an event rate of 32% (68% successful AF ablations) (4, 5), inclusion of 563 patients is required to achieve sufficient events. Accounting for a lost-to-follow-up rate of 15%, the aim is to include 650 patients scheduled for first catheter ablation.

Variables with possible interrelation (e.g., left atrial volume index or AF type with chosen ablation technique) will be tested for interaction and, if present, interaction terms will be included in the model. The model will be developed using logistic regression by forward selection with the use of the Akaike Information Criterion (AIC) as stopping rule. The discriminative performance of the model is evaluated by calculating the area under the receiver operator characteristic (AUROC) curve (C-statistic) and by comparisons of observed groups of different predictive frequencies. To assess the potential confounding effect of the ablation strategy that was chosen, performance analyses will be done for the entire cohort and separately for patients treated with different ablation strategies for sensitivity purposes.

### Current Study Status

The first ISOLATION patient was enrolled on 8 July 2020. On 1 January 2022, 405 patients have been included in the ISOLATION study (267 in the MUMC and 147 in the Radboudumc). In the same time period, 206 patients have been included in the ISOLATION “light” registry, and only 79 patients undergoing AF ablation did not consent to study participation (Supplementary Material 3). The intended number of 650 participants is expected to be reached in October 2022, with complete follow-up data in October 2024.

## Discussion

Despite the advances in ablation techniques and the increasing knowledge of AF mechanisms, ablation outcomes remain relatively poor (4). Improved patient selection for invasive management and a graded choice for the type of ablation could help to enhance success rates and avoid unnecessary procedures and associated risks. In the multicenter, prospective ISOLATION cohort study, a range of possible predictors for ablation success is investigated. As AF is a multifactorial disease with numerous possible underlying mechanisms, predictors for successful treatment can be identified in several different domains. Combining recognized predictors with newer techniques that take different disease mechanisms into account may improve patient selection strategies. For this purpose, several established predictors within the clinical domain (e.g., age and comorbidities) are collected and complemented with possible predictors from investigational methods such as anthropometric information (15). Preprocedural AF patterns, while traditionally only described as paroxysmal, persistent, or longstanding persistent, are further specified toward a more detailed pattern description concerning the duration and frequency of the episodes. Echocardiography-derived information on cardiac anatomy will be supplemented with findings from cardiac CT or CMR. Non-invasive electrophysiological information will incorporate predictors derived from extECGs, in addition to those from the standard 12-lead ECGs. Biomarkers reflecting inflammation, prothrombotic state and endothelial dysfunction are measured and related to AF ablation success and common gene variants are scrutinized to determine the predictive value of an individual’s genetic background.

Keeping in mind the daily clinical practice, it would be desirable to implement a limited number of additional modalities to improve patient selection. Therefore, results from different examinations will be grouped and compared to determine the most suitable variable(s) and modalities for outcome prediction. Identifying these variables which hold the largest predictive value may help to tailor treatment strategy and timing to the individual patient.

To achieve a clinically relevant, heterogeneous study population, the aim is to include a representative cohort of consecutive patients undergoing AF ablation in the two participating centers. Therefore, the in- and exclusion criteria are broad and the study is embedded in the pre-ablation work-up to ensure maximum efficiency for patients, clinicians, nurses, and researchers. The seamless integration of the informed consent procedure and research procedures in the clinical pathway reduces “missed inclusions” due to logistical reasons or lack of identification of eligible patients, and it decreases the additional time burden placed on participating patients. Patients who are still apprehensive of participation in ISOLATION are offered participation in the “light” version of the study, which omits almost all additional study procedures and primarily facilitates collection of data which are collected as part of clinical care. With this approach, almost 90% of consecutive AF ablation patients have been included in either of the studies in the first year of enrollment, providing a nearly complete representation of patients undergoing AF ablation in the two ablation centers.

Besides integrating clinical care and research, this pathway also provides the opportunity to integrate important components of AF management as recommended in the European Society of Cardiology (ESC) AF guidelines (3). All patients undergo structured risk factor assessment as part of the pre-AF ablation work-up, and in this context they are screened for hypertension, hyperlipidemia, diabetes mellitus, obesity, OSA, and structural heart disease. This approach makes it one of the first large cohorts with systematically assessed data on all classical comorbidities in AF patients, which are often underreported in other studies. Furthermore, it enables timely initiation of treatment of these risk factors. Patient engagement is encouraged by extensive education upon AF, risk factors, research participation, and the ablation procedure itself. The entire process, from patient education to management of comorbidities and research participation, is organized in an integrated, multidisciplinary care approach, overseen by a case manager, thus implementing all guideline-recommended pillars of AF care.

The described AF ablation pathway provides a research platform in which not only the primary aim is addressed, but which also offers opportunities to study numerous secondary objectives. Interventional or other observational substudies can easily be incorporated, either for the entire cohort or subgroups of patients. In addition, it is possible to include a randomization module into this observational study to realize a registry-based randomized clinical trial. This type of study is gaining popularity, as it combines the advantages of a prospective randomized trial (high level of evidence, strict control of confounding factors) with those of a large-scale all-comers clinical registry (less selection bias, fewer logistical challenges) (16). Questions regarding small process or treatment variations may be easily addressed with such a randomization module, without the disadvantage of constructing an entirely new logistical pathway with its associated costs. During the course of the study, several collaborations with (sub) specialties within our institutions have been established, such as cardiac imaging (e.g., evaluation of extent of fibrosis on cardiac magnetic resonance imaging before and after AF ablation, examination of flow patterns), translational cardiac electrophysiology (e.g., ECG-imaging, analysis of endocardial mapping signals), cardio-thoracic surgery (LA biopsies, epicardial mapping), pulmonology, and anesthesiology. This illustrates the role of ISOLATION as a crucial step toward the structural integration of high profile electrophysiological research in top referral clinical care pathways (17).

### Limitations

The ISOLATION study and ISOLATION “light” registry have several limitations. First, the type of AF ablation that is performed is not standardized but is decided upon by a patients’ treating physician. Success rates of different strategies vary and this may impact the rate in which the primary endpoint occurs. Second, monitoring for recurrences of atrial arrhythmia is performed intermittently using ECGs, Holter recordings, and PPG-based monitoring, but lack of continuous rhythm monitoring may lead to an underestimation of the number of endpoints (18). Third, only AF patients scheduled for AF ablation are included. Results found in this cohort may not be generalizable to the entire AF population. Fourth, not all patients undergoing AF ablation will agree to study participation. Although the integration with the ISOLATION “light” registry helps to include nearly all patients undergoing AF ablation, the specific study procedures are only conducted for participants in the main ISOLATION study. Fifth, the study reflects a large, real-world cohort of AF ablation patients. Due to the world-wide COVID-19 pandemic and to incident logistical reasons, protocol deviations may occur (e.g., a longer than desirable waiting time for ablation or out-of-window follow-up visits).

## Conclusion

This cohort study explores 6 different domains for predictors of successful AF ablation: (1) clinical factors, (2) AF patterns, (3) anatomical characteristics, (4) electrophysiological characteristics, (5) circulating biomarkers, and (6) genetic background. All study procedures are incorporated into a standardized, integrated clinical care and research pathway allowing an almost complete consecutive recruitment of patients undergoing AF ablation in two Dutch AF centers. The findings from different domains will be combined to determine the optimal combination of established predictors and novel techniques. This combination of predictors could then be used to tailor treatment decisions specifically to individual patients and to improve patient selection for invasive management.

## Ethics Statement

The studies involving human participants were reviewed and approved by METC azM/UM. The patients/participants provided their written informed consent to participate in this study.

## Author Contributions

DV, DL, SC, SW, DU, CH, BH, JT, AI, BM, KV, and US contributed to conception and design of the study. DV, DL, SC, TL, CH, EB, BH, SZ, GB, JH, RH, CM, RN, VE, CK, SS, WB, BM, KV, and US involved in conception and design of substudies of the ISOLATION. DV, DL, SC, SW, DU, SP, MK, ZH, BV, RMB, RJB, RE, MH, JL, RM, TL, CH, EB, BH, SZ, GB, SS, BM, and KV involved in data generation. DV, SP, MK, ZH, BV, CH, and GB involved in data management. DV wrote the first draft of the manuscript. All authors contributed to manuscript revision, read, and approved the submitted version.

## Conflict of Interest

CM received consultancy fees for Bayer Healthcare. SS received grants from Boehringer Ingelheim, AstraZeneca and GlaxoSmithKline, honorary fees for presentations from AstraZeneca and Chiesi, consultancy fees from GlaxoSmithKline, payment for advisory boards from Chiesi and GlaxoSmithKline, all outside the submitted work, and payed to his institution. KV was consultant for Medtronic, Abbott, Philips, Biosense Webster. US received consultancy fees or honoraria from Università della Svizzera Italiana (USI, Switzerland), Roche Diagnostics (Switzerland), EP Solutions Inc. (Switzerland), Johnson & Johnson Medical Limited (United Kingdom), Bayer Healthcare (Germany). US was co-founder and shareholder of YourRhythmics BV, a spin-off company of the University Maastricht. The remaining authors declare that the research was conducted in the absence of any commercial or financial relationships that could be construed as a potential conflict of interest.

## Publisher’s Note

All claims expressed in this article are solely those of the authors and do not necessarily represent those of their affiliated organizations, or those of the publisher, the editors and the reviewers. Any product that may be evaluated in this article, or claim that may be made by its manufacturer, is not guaranteed or endorsed by the publisher.
