# Supplementary material for: Rationale and Design of the ISOLATION Study: A Multicenter Prospective Cohort Study Identifying Predictors for Successful Atrial Fibrillation Ablation in an Integrated Clinical Care and Research Pathway
Source: Front Cardiovasc Med. 2022 Jun 30;9:879139. doi: 10.3389/fcvm.2022.879139 (PMC9307503; doi:10.3389/fcvm.2022.879139)
Supplement: Supplementary file 1 [file Data_Sheet_1.docx]

****SUPPLEMENTS****

# **Supplement 1. Description and discussion of study procedures**

**The following procedures are performed as additional study measurements for all participants in the ISOLATION study: determination of body composition, pre-procedural rhythm monitoring, extended surface electrocardiogram (extECG), biomarker testing, genetic analysis, and questionnaires.**

## Determination of body composition

Although obesity is a well-known risk factor for atrial fibrillation (AF) development and AF burden, recent studies have indicated that obesity-related anthropometric measures such as fat percentage and total body mass index are not associated with AF development after adjusting for lean body mass.^(1)^ To explore whether the same association is present with the development of recurrences after AF ablation, this study will examine the correlation between different anthropometric measures (**Table S1**) and ablation success. Weight, fat percentage and lean body mass indices will be estimated using previously developed and validated^(2)^ sex-specific equations on bioelectrical impedance, measured with body composition monitors (Omron BF511 Body Composition monitor, Omron Healthcare). The results of these measurements are part of domain 1: clinical factors.

## Pre-procedural rhythm monitoring

The predictive value of different AF patterns for successful AF ablation has not been previously studied. However, this domain may yield interesting information. Frequent, short AF episodes may identify patients with frequent triggers initiating AF, while longer sustained AF episodes may identify those with pronounced atrial structural remodeling maintaining AF. As pulmonary vein isolation (PVI) targets AF triggers originating from the pulmonary veins (PVs), it is thus expected to be more successful in patients who predominantly suffer from frequent, short paroxysms. Indeed, ablation success rates of PVI only are higher for patients with paroxysmal AF than for those with persistent AF,^(3)^ who often have more extensive LA fibrosis potentially harboring extra-PV triggers for AF. However, many more AF phenotypes exist within the groups of paroxysmal and persistent AF. A more detailed classification of AF patterns taking frequency and duration of separate paroxysms into account is therefore desirable.^(4)^ To gain more insight in these patterns, ISOLATION patients awaiting the AF ablation procedure receive a handheld patient-operated device (MyDiagnostick 1001R, Applied Biomedical Systems, Maastricht, the Netherlands) which records and stores one-lead ECGs. Patients are asked to record their heart rhythm three times a day during a maximum of four weeks. Additional registrations are recorded at each onset or relief of arrhythmia symptoms. Symptoms and their correlation to measurements are recorded in a patient diary. The AF patterns are studied in domain 2 (**Table S1**).

## Extended surface electrocardiogram

Detailed analysis of P-wave and fibrillation wave (F-wave) features from surface electrocardiograms (ECGs) are increasingly used to characterize the degree of electrophysiological changes in the atria. Several P-wave characteristics (e.g. P-wave duration, amplitude, area, and force^(5-7)^) and fibrillation wave (F-wave) characteristics (e.g. dominant frequency, amplitude, and organization index^(8-12)^) have been demonstrated to predict response to rhythm control strategies. ECG recordings using additional leads that specifically focus on

**Table S1. Variables of interest in the ISOLATION study in domain 1-5**

| **Parameters of interest per domain** | | | | |
| --- | --- | --- | --- | --- |
| **Domain 1: Clinical factors** | | | | |
| Age | Congestive heart failure | | Body mass index | |
| Sex | Previous thromboembolic events | | Fat percentage | |
| **Comorbidities** | Sleep apnea | | Fat mass | |
| Hypertension | Smoking status | | Visceral fat level | |
| Diabetes mellitus | Alcohol consumption | | Muscle percentage | |
| Dyslipidemia | **Anthropometric information** | | Muscle mass | |
| Coronary artery disease | Height | | Fat-free mass (lean mass) | |
| Vascular disease | Weight | | Basal metabolic rate | |
| **Domain 2: Pre-procedural AF patterns** | | | | |
| AF type | N^o^ of non-AF episodes | | Maximum heart rate in AF | |
| N^o^ of AF episodes | Median duration non-AF episodes | | Median heart rate in SR | |
| Median duration of AF episodes | Percentage of time in AF | | AF pattern classification | |
| Duration of longest AF episode | Predominant onset of AF in morning/afternoon/evening/night | |  | |
| Duration of shortest AF episode | Median heart rate in AF | |  | |
| **Domain 3: Anatomical characteristics** | | | | |
| Left ventricular ejection fraction | Left atrial diameter | | N^o^ of right-sided pulmonary veins | |
| Left ventricular end-diastolic dimension | Left atrial volume | | N^o^ of left-sided pulmonary veins | |
| Left ventricular mass | Left atrial volume index | | Agatson calcium score | |
| Left ventricular mass index | Right atrial area | | Presence of coronary plaques | |
| Mean E/e’ | Right atrial volume | |  | |
| Right ventricular systolic pressure | N^o^ of pulmonary veins | |  | |
| **Domain 4: Electrophysiological characteristics** | | | | |
| Rhythm | | Terminal force | | f-wave amplitude |
| Heart rate | | Shannon entropy | | Organization index |
| QRS duration | | Sample entropy | | Regularity index |
| QT duration | | P-wave complexity | | Spectral entropy |
| QTc interval | | Euclidean distance | | Sample entropy |
| **P-wave characteristics** | | Similarity index | | f-wave power |
| P-wave duration | | Spatial similarity | | Harmonic decay |
| P-wave area | | **F-wave characteristics** | | N^o^ of principal components |
| P-wave amplitude | | Dominant frequency | | Spatiotemporal organization |
| **Domain 5: Circulating biomarkers** | | | | |
| Hemoglobin | Endothelial cell-specific molecule 1 | | Dickkopf-related protein 3 | |
| Creatinine | Pro-brain natriuretic peptide 2 | | Insulin-like growth factor-binding protein 7 | |
| Interleukin 6 | N-terminal pro-BNP | | Bone morphogenetic protein 10 | |
| Angiopoietin 2 | Fibroblast growth factor 23 | |  | |

*Variables of interest collected in the ISOLATION study and ISOLATION ‘light’ registry. In case of newly gained insights during the course of the study, the list may be supplemented with additional parameters. AF: atrial fibrillation, BNP: brain natriuretic peptide, ECG: electrocardiogram, f-wave: fibrillation wave, QTc: corrected QT-interval, SR: sinus rhythm*

the atria may provide even more information.^(13-15)^ In the ISOLATION study, extECGs are recorded at three separate time points. Eleven additional electrodes are placed on the front and the back of the patient (**Figure S1**), besides the ten electrodes used to acquire the standard 12-lead ECG. Eight of the additional electrodes are placed to construct a cuboid box from which 3D vectorcardiograms can be calculated. Three electrodes are placed in positions focusing on signals from the left and right atrium. The extECG will be recorded unfiltered for up to five minutes to acquire about 300 P-waves (in the case of sinus rhythm) or 1500 to 2000 fibrillation waves (in the case of AF). The longer recordings allow for signal-averaged analyzation of P-waves and for QRST cancellation to examine F-wave frequency and complexity dynamics with minimum artifacts. ExtECGs are recorded using the YRS100 cardiac amplifier (YourRhythmics BV, Maastricht, The Netherlands). Variables of interest include those listed in **Table S1** and are studied in domain 4: electrophysiological characteristics.

****
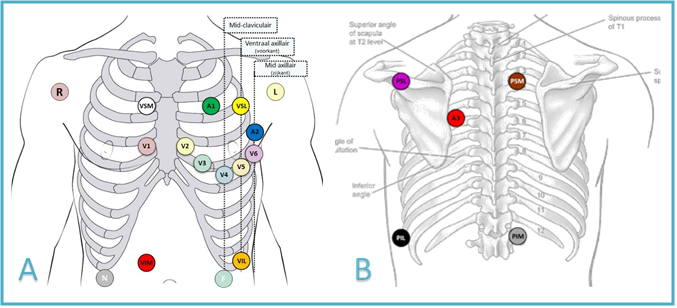
**Figure S1. Placement leads extended surface electrocardiogram**

***Placement of the electrodes of the extended surface electrocardiogram at the front (A) and the back (B) of the patient.*** *Eight electrodes are placed to construct a cuboid box (VSM, VSL, VIL, VIM, PSM, PSL, PIL, PIM). Three electrodes are placed in positions focusing on signals from the left and right atrium (A1, A2, A3). The remaining 10 electrodes (R, L, F, N, V1, V2, V3, V4, V5, V6) are placed in the usual positions for standard 12-lead electrocardiograms.*

## Blood samples

Several biomarkers and common gene variants have been identified that predict AF development, AF-related complications, and recurrences of AF after ablation.^(16-20)^ The biomarkers of interest that are studied in the ISOLATION and ISOLATION ‘light’ studies are listed in **Table S2** and include inflammatory mediators,^(17)^ markers of myocardial wall stress,^(17)^ markers of fibrosis,^(21, 22)^ markers of endothelial dysfunction and pro-thrombotic state,^(23-25)^ and markers for expression of genes regulating electrophysiological characteristics.^(16)^ In addition, the results of several ongoing trials collecting a broad set of biomarkers and genetic data in large cohorts will be taken into account in this study’s analysis.^(26, 27)^ Blood for biomarker analyses is drawn at three separate time points. Genetic analysis is performed on the blood drawn at baseline. The procedures for the collection, processing and storage of blood samples are described in a standardized operating procedure to assure maximum quality and comparability of the samples. Frozen samples will be shipped periodically to external laboratories for biomarker analyses (Roche – Roche Diagnostics Rotkreuz, Switzerland) and genetic analysis (University Hospital Münster, Germany, Department of human genetics and genetic epidemiology). The results are studied in domain 5: circulating biomarkers and 6: genetic background.

**Table S2. Biomarkers of interest**

| **Biomarker/genetic background** | **Abbreviation** | **Category** |
| --- | --- | --- |
| Interleukin 6 | IL-6 | Inflammatory mediator |
| Angiopoietin 2 | ANG-2 | Inflammatory mediator, marker of endothelial disfunction |
| Endothelial cell-specific molecule 1 | ESM-1 | Marker of endothelial disfunction |
| Pro-brain natriuretic peptide 2 | pro-BNP2 | Marker of myocardial wall stress |
| N-terminal pro-brain natriuretic peptide | NT-proBNP | Marker of myocardial wall stress |
| Fibroblast growth factor 23 | FGF-23 | Marker of fibrosis |
| Dickkopf-related protein 3 | DKK-3 | Marker of fibrosis |
| Insulin-like growth factor-binding protein 7 | IGFBP-7 | Marker of pro-thrombotic state |
| Bone morphogenetic protein 10 | BMP-10 | Marker for expression of genes regulating electrophysiological characteristics |
| Transcription factor PITX2 | PITX2 | Transcription factor involved in the development of the left and right atria and pulmonary veins |

*Several biomarkers of interest in the ISOLATION study and ISOLATION ‘light’ registry. In the case of new insights during the course of the studies, the list may be supplemented with additional biomarkers of interest.*

## Questionnaires

The following questionnaires are completed at baseline: Montreal Cognitive Assessment (MoCA), Toronto AF Severity Scale (AFSS), Atrial Fibrillation Effect on QualiTy-of-life (AFEQT), STOP-Bang, and a combined COPD questionnaire consisting of a modified Respiratory Health Screening Questionnaire (RHSQ), COPD assessment test (CAT), and modified Medical Research Council (mMRC) Dyspnea scale. The MoCA test is completed on site with the help of trained study personnel. The AFSS, AFEQT, STOP-Bang, and combined COPD questionnaire are completed using either paper versions or electronic versions, depending on patient preference. Additional information on the questionnaires is provided in **Table S3**.

## **Study procedures for subsets of patients**

The **following procedures are performed as additional study measurements in a subset of ISOLATION participants: trans-esophageal electrocardiogram (TE-ECG), additional analysis of endo- or epicardial mapping, and left atrial appendage (LAA) biopsies.**

**Transesophageal electrocardiogram**

**Participants are asked for separate permission to record a transesophageal ECG. The TE-ECG is recorded using a catheter containing multiple unipolar leads. This catheter is either swallowed or inserted through the nostril, depending on the researchers and patient’s preference, to reach the esophagus. It is held in place to record for 2 minutes, or as long as tolerated, and is then removed.**

Table S3. Questionnaires

|  | **Prior to ablation (baseline)** | **3 months after ablation** | **12 months after ablation** | **24 months after ablation** |
| --- | --- | --- | --- | --- |
| **Toronto AF Severity Scale (AFSS)**  *Questionnaire on type and intensity of AF-related symptoms*  *19 questions* | | | | |
| **Timing** | X | X | X | X |
| **Atrial Fibrillation Effect on QualiTy-of-life (AFEQT)**  *Assessment of quality of life specifically for AF patients*  *22 questions* | | | | |
| **Timing** | X | X | X | X |
| **Montreal Cognitive Assessment (MoCA)**  *Assessment of cognitive impairment in multiple cognitive domains*  *30 questions* | | | | |
| **Timing** | X | X | X |  |
| **Stop-Bang**  *Risk estimation for obstructive sleep apnea*  *8 questions* | | | | |
| **Timing** | X |  |  |  |
| **Combined COPD questionnaire***  *Assessment of symptoms of COPD and its impact on the patient’s health*  *15 questions* | | | | |
| **Timing** | X |  |  |  |

*Timing and purpose of questionnaires in the ISOLATION study. * The combined COPD questionnaire includes a modified Respiratory Health Screening Questionnaire (RHSQ), COPD assessment test (CAT), and modified Medical Research Council (mMRC) Dyspnea scale. COPD = chronic obstructive pulmonary disease.*

**Endo- or epicardial mapping**

**Depending on the selected ablation technique, endocardial or epicardial electroanatomical mapping may be performed. Epicardial mapping is performed as additional study procedure, endocardial mapping is standard practice in some transcatheter endocardial procedures. When performed, results of epi- and endocardial mapping are saved to use for additional analysis in a later stadium. Custom-made algorithms will be used to identify activation times based on electrograms and to reconstruct waves that inform on the predominant activation pattern and conduction velocity during sinus rhythm, AF and routine atrial pacing. These activation patterns will be correlated to the electrogram properties that have been used for individualized and targeted ablation (e.g. low voltage areas, high degree of electrogram fractionation). This investigation will deepen our understanding on the mechanism of AF termination by ablation of such areas. Besides, a newly developed algorithm to detect repetitive conduction patterns and reconstruction of macro-reentry circuits based on these patterns will be used and further refined. This algorithm will allow to determine critical paths for re-entry that independently from electrogram morphology may constitute a target for AF ablation.**

**Biopsy of left atrial appendage (LAA)**

**During hybrid ablations or concomitant surgical ablations, the LAA is usually clipped to reduce the risk of thromboembolisms. After the structure is clipped, a biopsy can be taken easily and safely. Studying this tissue will provide additional information about structural changes and differences in electrical conduction in the atria.**

**Supplement references**

1. Fenger-Gron M, Overvad K, Tjonneland A, Frost L. Lean Body Mass Is the Predominant Anthropometric Risk Factor for Atrial Fibrillation. J Am Coll Cardiol. 2017;69(20):2488-97.

2. Heitmann BL. Prediction of body water and fat in adult Danes from measurement of electrical impedance. A validation study. Int J Obes. 1990;14(9):789-802.

3. Ganesan AN, Shipp NJ, Brooks AG, Kuklik P, Lau DH, Lim HS, et al. Long-term outcomes of catheter ablation of atrial fibrillation: a systematic review and meta-analysis. J Am Heart Assoc. 2013;2(2):e004549.

4. Chen LY, Chung MK, Allen LA, Ezekowitz M, Furie KL, McCabe P, et al. Atrial fibrillation burden: moving beyond atrial fibrillation as a binary entity: a scientific statement from the american heart association. Circulation. 2018;137(20):e623-e44.

5. Blanche C, Tran N, Rigamonti F, Burri H, Zimmermann M. Value of P-wave signal averaging to predict atrial fibrillation recurrences after pulmonary vein isolation. Europace. 2013;15(2):198-204.

6. Kanzaki Y, Inden Y, Ando M, Kamikubo Y, Ito T, Mizutani Y, et al. An ECG index of P-wave force predicts the recurrence of atrial fibrillation after pulmonary vein isolation. Pacing Clin Electrophysiol. 2016;39(11):1191-7.

7. Rasmussen MU, Kumarathurai P, Fabricius-Bjerre A, Larsen BS, Dominguez H, Davidsen U, et al. P-wave indices as predictors of atrial fibrillation. Ann Noninvasive Electrocardiol. 2020;25(5):e12751.

8. Nault I, Lellouche N, Matsuo S, Knecht S, Wright M, Lim KT, et al. Clinical value of fibrillatory wave amplitude on surface ECG in patients with persistent atrial fibrillation. J Interv Card Electrophysiol. 2009;26(1):11-9.

9. Cheng Z, Deng H, Cheng K, Chen T, Gao P, Yu M, et al. The amplitude of fibrillatory waves on leads aVF and V1 predicting the recurrence of persistent atrial fibrillation patients who underwent catheter ablation. Ann Noninvasive Electrocardiol. 2013;18(4):352-8.

10. Matsuo S, Lellouche N, Wright M, Bevilacqua M, Knecht S, Nault I, et al. Clinical predictors of termination and clinical outcome of catheter ablation for persistent atrial fibrillation. J Am Coll Cardiol. 2009;54(9):788-95.

11. Lankveld T, Zeemering S, Scherr D, Kuklik P, Hoffmann BA, Willems S, et al. Atrial fibrillation complexity parameters derived from surface ECGs predict procedural outcome and long-term follow-up of stepwise catheter ablation for atrial fibrillation. Circ Arrhythm Electrophysiol. 2016;9(2):e003354.

12. Zeemering S, Lankveld TAR, Bonizzi P, Limantoro I, Bekkers S, Crijns H, et al. The electrocardiogram as a predictor of successful pharmacological cardioversion and progression of atrial fibrillation. Europace. 2018;20(7):e96-e104.

13. Meo M, Zarzoso V, Meste O, Latcu DG, Saoudi N. Spatial variability of the 12-lead surface ECG as a tool for noninvasive prediction of catheter ablation outcome in persistent atrial fibrillation. IEEE Trans Biomed Eng. 2013;60(1):20-7.

14. Meo M, Zarzoso V, Meste O, Latcu DG, Saoudi N. Non-invasive prediction of catheter ablation outcome in persistent atrial fibrillation by exploiting the spatial diversity of surface ECG. Annu Int Conf IEEE Eng Med Biol Soc. 2011;2011:5531-4.

15. Zink MD, Laureanti R, Hermans BJM, Pison L, Verheule S, Philippens S, et al. Extended ECG Improves Classification of Paroxysmal and Persistent Atrial Fibrillation Based on P- and f-Waves. Front Physiol. 2022;13:779826.

16. Reyat JS, Chua W, Cardoso VR, Witten A, Kastner PM, Kabir SN, et al. Reduced left atrial cardiomyocyte PITX2 and elevated circulating BMP10 predict atrial fibrillation after ablation. JCI Insight. 2020;5(16):e139179.

17. Vilchez JA, Roldan V, Hernandez-Romero D, Valdes M, Lip GY, Marin F. Biomarkers in atrial fibrillation: an overview. Int J Clin Pract. 2014;68(4):434-43.

18. Andersen JH, Andreasen L, Olesen MS. Atrial fibrillation-a complex polygenetic disease. Eur J Hum Genet. 2021;29(7):1051-60.

19. Choi SH, Weng LC, Roselli C, Lin H, Haggerty CM, Shoemaker MB, et al. Association between titin loss-of-function variants and early-onset atrial fibrillation. JAMA. 2018;320(22):2354-64.

20. van Ouwerkerk AF, Hall AW, Kadow ZA, Lazarevic S, Reyat JS, Tucker NR, et al. Epigenetic and transcriptional networks underlying atrial fibrillation. Circ Res. 2020;127(1):34-50.

21. Liu B, Li X, Zhao C, Wang Y, Lv M, Shi X, et al. Proteomic analysis of atrial appendages revealed the pathophysiological changes of atrial fibrillation. Front Physiol. 2020;11:573433.

22. Chen JM, Zhong YT, Tu C, Lan J. Significance of serum fibroblast growth factor-23 and miR-208b in pathogenesis of atrial fibrillation and their relationship with prognosis. World J Clin Cases. 2020;8(16):3458-64.

23. Bontekoe J, Lee J, Bansal V, Syed M, Hoppensteadt D, Maia P, et al. Biomarker profiling in stage 5 chronic kidney disease identifies the relationship between angiopoietin-2 and atrial fibrillation. Clin Appl Thromb Hemost. 2018;24(9_suppl):269S-76S.

24. Dudink EA, Weijs B, Tull S, Luermans JG, Fabritz L, Chua W, et al. The biomarkers NT-proBNP and CA-125 are elevated in patients with idiopathic atrial fibrillation. Journal of atrial fibrillation. 2018;11(4):2058.

25. Blum S, Aeschbacher S, Meyre P, Kuhne M, Rodondi N, Beer JH, et al. Insulin-like growth factor-binding protein 7 and risk of congestive heart failure hospitalization in patients with atrial fibrillation. Heart Rhythm. 2021;18(4):512-9.

26. Chua W, Easter CL, Guasch E, Sitch A, Casadei B, Crijns H, et al. Development and external validation of predictive models for prevalent and recurrent atrial fibrillation: a protocol for the analysis of the CATCH ME combined dataset. BMC Cardiovasc Disord. 2019;19(1):120.

27. Gilbers MD, Bidar E, Maesen B, Zeemering S, Isaacs A, Crijns H, et al. Reappraisal of Atrial fibrillation: interaction between hyperCoagulability, Electrical remodelling and Vascular destabilisation in the progression of AF (RACE V) Tissue Bank Project: study design. Neth Heart J. 2021;29(5):280-7.

28. Verhaert DVM, Betz K, Gawalko M, Hermans ANL, Pluymaekers N, van der Velden RMJ, et al. A VIRTUAL Sleep Apnoea management pathway For the work-up of Atrial fibrillation patients in a digital Remote Infrastructure: VIRTUAL-SAFARI. Europace. 2022;24(4):565-75.

# **Supplement 2.** Substudies of the ISOLATION and ISOLATION ‘light’ registry.

| **Subject** | **Subset of patients** | **Primary aims** |
| --- | --- | --- |
| **Transesophageal**  **ECG** | RF ablation (incl. electroanatomical mapping) | - To investigate the correlation between atrial depolarization waves measured with transesophgeal ECG and the real activation patterns obtained from invasive electroanatomic mapping. - To explore the association between f-wave characteristics and P-wave characteristics derived from transesophgeal ECG with arrhythmia recurrence. |
| **ECG-imaging** | RF ablation (incl. electroanatomical mapping) | - To investigate whether atrial depolarization waves measured with ECGi accurately reflect the real activation patterns obtained from invasive electroanatomic mapping. - To investigate the relation between activation patterns obtained from ECGi with clinical parameters (e.g. paroxysmal vs persistent, left atrial size, recurrences). |
| **CMR**  *C-SUBSTRATE* | RF ablation (incl. electroanatomical mapping) | - To investigate left atrial substrate for AF with innovative cardiac CMR techniques. - To investigate the effect of AF ablation on left atrial substrate by evaluating anatomical, hemodynamic and functional changes by CMR. - To explore the association between left atrial substrate (in 3 domains) and arrhythmia recurrence |
| **LAA biopsy**  **Epicardial electroanatomical mapping** | Surgical ablation or hybrid ablation | - To study structural changes and differences in electrical conduction in the atria by determining overall connective tissue content, endomysial fibrosis, capillary density and fibroblast abundance. - To unravel molecular pathways associated with AF or recurrences of AF by mRNA sequencing - To study AF mechanisms measured by epicardial mapping and to relate the AF conduction patterns to the underlying atrial tissue characteristics |
| **Concomitant hybrid AF ablation**  *COMBAT-AF* | CABG + concomitant AF | - To evaluate the feasibility, safety and effectivity of a staged hybrid concomitant treatment in patients with persistent AF that undergo a CABG and did not have prior AF ablations. - To evaluate the potential of non-invasive parameters to predict the completeness of surgical ablation lines - To study the underlying AF mechanism based on the epicardial and endocardial mapping and to relate the AF conduction patterns to the underlying atrial tissue characteristics. |
| **OSA screening**  ***VIRTUAL-SAFARI*** *^(28)^* | All patients | - To evaluate the feasibility of structural, remote OSA screening in patients awaiting AF ablation - To assess the prevalence of previously undiagnosed OSA in patients awaiting AF ablation - To explore the impact of structural OSA screening and treatment on AF ablation outcomes and symptoms |
| **COPD screening** | MUMC+ patients | - To evaluate the feasibility of structural COPD screening using handheld spirometry in patients awaiting AF ablation - To assess the prevalence of previously undiagnosed COPD in patients awaiting AF ablation - To explore the impact of structural COPD screening and treatment on AF ablation outcomes and symptoms |

*Legend: Table listing the different substudies incorporated in the ISOLATION study and ISOLATION ‘light’ registry. AF = atrial fibrillation, CABG = coronary artery bypass grafting, CMR = cardiac magnetic resonance imaging, COPD = chronic obstructive pulmonary disease, ECG = electrocardiogram, ECGi = electrocardiographic imaging, LAA = left atrial appendage, mRNA = messenger ribonucleic acid, MUMC+ = Maastricht University Medical Center, OSA = obstructive sleep apnea syndrome, RF = radiofrequency energy.*

# **Supplement 3.** Inclusion in ISOLATION study and ISOLATION ‘light’ registry

**
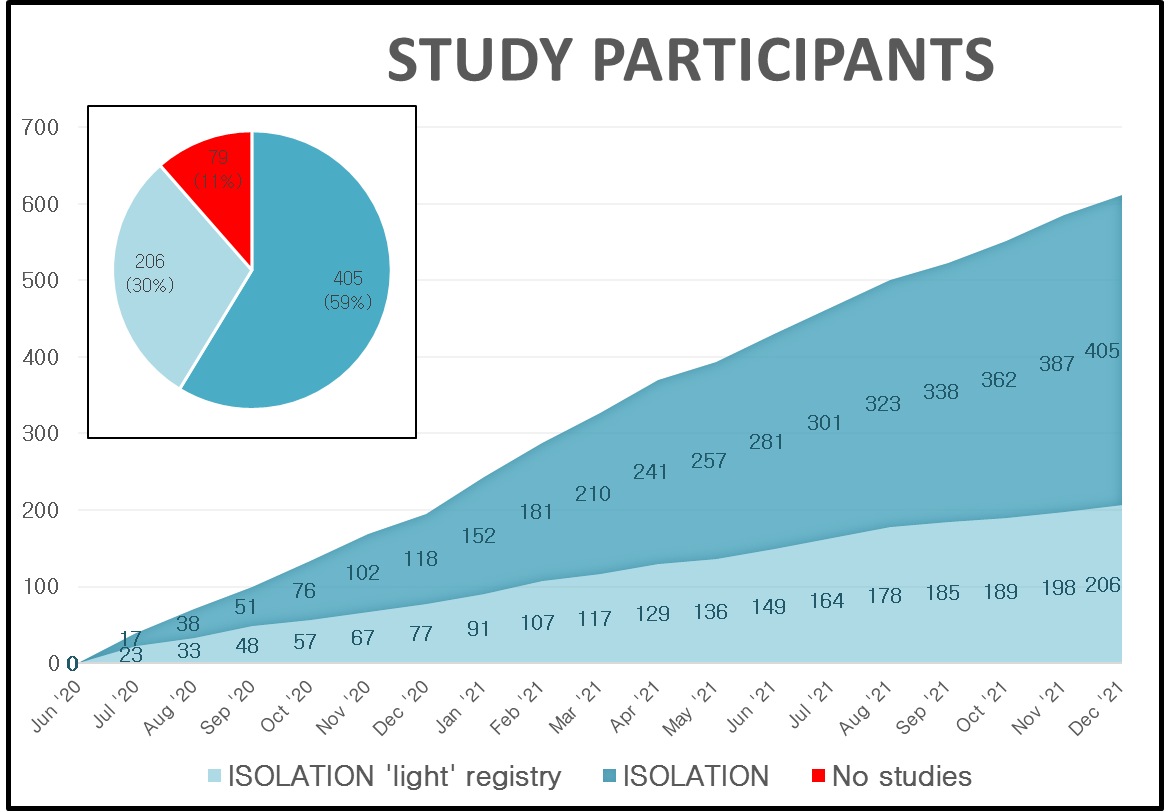
**

*Legend: Inclusion rates in the ISOLATION study and the ISOLATION ‘light’ registry over time from June 2020 until December 2021. The pie chart in the upper left corner displays the percentages of all patients undergoing atrial fibrillation ablation in the two participating centers that was included in the ISOLATION study (59%), in the ISOLATION ‘light’ registry (30%) and patients that declined participation in either study (only 11%).*
